# Use of Biological Treatments in Elderly Patients with Skin Psoriasis in the Real World

**DOI:** 10.3390/life11121348

**Published:** 2021-12-07

**Authors:** Cristina Galache Osuna, Sebastián Reyes García, Jimena Carrero Martín, Virginia García Jiménez, Francisco Vázquez López, Jorge Santos-Juanes

**Affiliations:** 1 Dermatology, Hospital Universitario Central de Asturias, 33011 Oviedo, Asturias, Spain; cristinagalache@gmail.com (C.G.O.); sebastian021314@gmail.com (S.R.G.); jimenacarrerom@gmail.com (J.C.M.); vazquez@uniovi.es (F.V.L.); 2 Clinical Management Unit, UGC Farmacia, Hospital Universitario Central de Asturias, 33011 Oviedo, Asturias, Spain; virginia.garciaj@sespa.es; 3 Dermatology, Instituto de Investigación Sanitaria del Principado de Asturias, IUOPA, University of Oviedo, 33011 Oviedo, Asturias, Spain; 4 Spanish Biomedical Research Network Centre in Oncology, CIBERONC, Av. Monforte de Lemos, 3-5, Pabellón 11, Planta 0, 28029 Madrid, Madrid, Spain

**Keywords:** psoriasis, elderly, biological treatment, drug survival

## Abstract

Biological drugs have prompted a revolution in the treatment of patients with psoriasis because of their favourable efficacy/risk profile. The aims of our study are to determine whether there is any difference in the pattern of use of biological treatments for older (65+ years) and younger patients diagnosed with plaque psoriasis by the Dermatology Service of the Hospital Universitario de Asturias (HUCA), to understand the survival of these drugs, and to identify the factors that predict the discontinuation of treatments. We report a retrospective observational hospital-based study of 300 patients registered at HUCA’s Dermatology Service who were receiving one of the following biological treatments for psoriasis on 30 November 2020: adalimumab, ustekinumab, secukinumab, or ixekizumab. The age groups were compared using Student’s t-test for quantitative variables and the chi-squared test for qualitative variables. We used the Kaplan–Meier estimator to estimate the survival function and the log-rank test to measure differences. No statistically significant differences in the frequency of use were noted between the younger and older groups, for any of the drugs studied. Survival on a drug regime, globally and individually, was similar in the two age groups. Factors predicting lower overall survival were being female, obesity, and having undergone previous biological treatment. The first three factors were influential in the under-65-year-old group, while arthritis was a significant factor for the older group.

## 1. Introduction

Psoriasis is a chronic inflammatory disease of the skin [1]. It has a multifactorial aetiology in which the genetic susceptibility of an individual interacts with environmental factors to produce dysregulation of the immune system [2]. It affects approximately 1–3% of the world’s population. Its prevalence varies by geographic location and ethnicity and increases with age due to the increase in life expectancy but does not differ between the sexes [3]. It can be diagnosed at any age, but there are two periods of peak incidence: one at 15–30 years and the other at 50–60 years [4].

At present, it is considered a systemic disease with predominantly cutaneous manifestations [5,6].

In recent years, it has become clear that psoriasis patients have higher incidences of cerebral and cardiovascular diseases and tend to die younger than healthy individuals [7,8]. Psoriasis is epidemiologically associated with diseases whose shared chronic inflammation is a common pathogenic substrate. Associated comorbidities or diseases usually manifest themselves years after the onset of the disease and appear more frequently in patients with severe psoriasis [9]. It has been linked to Crohn’s disease, metabolic syndrome and all its components, liver steatosis (non-alcoholic fatty liver disease), pulmonary involvement, depression, bipolar disorder, anxiety, addictions such as alcoholism and smoking, lymphomas, and solid tumours, and, more recently, changes in renal function [10,11,12].

Psoriatic arthritis is present in 5–30% of these patients, being most prevalent in those with more severe cutaneous psoriasis. It is a chronic inflammatory disease of the musculoskeletal system that is also mediated by the immune system. It affects both sexes equally, and usually occurs after skin involvement [13,14].

Psoriasis is a chronic pathology for which there is currently no curative treatment, which is why the aim of improving skin lesions, and thereby quality of life, is pursued. Most patients need long-term therapies for good disease control [15]. There are several options for treating psoriasis. We can distinguish between topical therapy, phototherapy and systemic therapy, within which conventional and biological agents are included [16]. The change of treatment, and the combinations of several agents, are common practices in the management of this disease, whose main aim is to reduce the cumulative toxicity of the drugs. Decisions regarding treatment of psoriasis are taken on an individual basis, considering the severity of psoriasis, the presence or absence of arthritis and other comorbidities, patient age, life expectancy, and possible adverse effects, among others [17].

It is accepted that older age in Western countries is defined as beginning at 65 years [18]. Information about treatments for elderly individuals is scarce and the management of these patients can be difficult due to the comorbidities they present and the interactions between the drugs they are usually prescribed [4].

Older patients tend to have more comorbidities, such as obesity, diabetes, dyslipidaemia, and cardiovascular diseases, among others [4], psoriatic arthritis being of particular relevance [19]. Treatment with other drugs is also widespread, and this carries the risk of pharmacological interaction and the worsening of baseline psoriasis [20]. In addition, many of these patients present what is known as immunosenescence, the reduced activity of the immune system, which makes them likely to exhibit adverse effects such as infections and cancer [21]. Age also leads to changes in pharmacokinetics: lower absorption and hepatic metabolic capacity, and impaired renal clearance. The latter is of great importance and should be considered when scheduling treatments [20].

Older patients with psoriasis can often be under-treated due to the problems posed. Topical therapy is the first choice because it has adverse effects, but phototherapy is a widely used alternative [21]. In general, systemic conventional and biological drugs have been less frequently used in older than in younger patients, possibly because the older patients have lower Psoriasis Area and Severity Index (PASI) scores than those in younger age groups; furthermore, for the same PASI, the Dermatology Life Quality Index (DLQI) is usually lower in older patients. Conventional systemic drugs, such as methotrexate, acitretin and cyclosporine, tend to be avoided due to their high toxicity in the elderly.

It is important to carry out many physical and analytical controls in the follow-up of patients treated with systemic drugs, and to make dose adjustments when required [22].

Biological drugs have led to an improvement in the prognosis of psoriasis and are increasingly used [16]. They have demonstrated a favourable efficacy/risk profile in several clinical studies and in post-marketing surveillance. They are indicated when established topical and systemic therapies fail, are not tolerated, or are contraindicated. Moderate–severe psoriasis (PASI ≥ 10, BSA ≥ 10%, DLQI ≥ 10), pustular psoriasis or psoriasis in locations that negatively affect the patient’s life (e.g., on the face, palms and soles, or in the genital region) are included [17]. We distinguish TNFα antagonists, IL-12/23 inhibitors, IL-23 inhibitors, and 17 inhibitors by their mechanism of action [16,23].

The longer-established treatments have their limitations, mainly due to their range of side effects, but biological treatments are not exempt from these, either [15]. The adverse effects (e.g., opportunistic infections, reactivation of tuberculosis, higher incidence of tumours, etc.) derive mainly from their mechanism of action as inhibitors of the immune system, but there are others that are specific to each molecule [24].

The survival of a drug is the duration of a specific therapy, taken as the period during which that particular drug remains an appropriate option for a specific patient [25]. It is conditioned by the maintenance or suspension of therapy, mainly for reasons of safety or due to loss of efficacy. All studies suggest that biological therapies have a favourable profile with respect to their safety [14].

If we accept that biological therapies for psoriasis will not, in most cases, be suspended for safety reasons, then the effectiveness or clinical response will be the main factor requiring analysis [26].

Other authors similarly interpret drug survival as a marker of therapeutic success since it depends on the efficacy, the presence of adverse reactions and patients’ satisfaction with their treatment.

Unlike clinical trials, analyses of biological therapy survival in psoriasis provide us with estimates of the efficacy of drugs over time in real clinical practice, in which doses are adjusted according to the evolution of the disease and in which adjuvant treatments are administered. Patient satisfaction is a major factor in the decision to introduce a therapeutic change.

This type of analysis in clinical practice can be influenced by changes in the prices of biological treatments, the appearance of new drugs and changes in prescription habits. For these reasons, it has been proposed that comparisons between survival studies of different drugs should be made with great caution [27]. In various pharmacovigilance studies of biological drugs, age does not seem to be highly related to an increase in side effects [28].

When choosing between a conventional and a biological systemic drug, biological drugs tend to be preferred because they are targeted [21], which increases their efficacy and reduces their adverse effects, especially the risk of infection [19]. There is no evidence that these side effects increase significantly with age [20].

Of the biological drugs, those of the anti-TNFα type are generally the first option. They have been shown to improve the PASI and DLQI, irrespective of age group [29]. Secukinumab, ustekinumab, and ixekizumab have also been used, and found to have a good safety profile and a high degree of effectiveness. Novel anti-IL 23 drugs, such as risankizumab, have been introduced to the market and promise an effectiveness similar to anti-IL 17 drugs [30].

We aimed to determine and quantify any differences in the pattern of use of four biological drugs—adalimumab, ustekinumab, secukinumab, and ixekizumab—establish whether the survival of the drugs is different in older patients and to study the factors that predict drug discontinuation in older (65+ years) and younger (under 65 years) patients.

## 2. Materials and Methods

We designed a retrospective, hospital study to be carried out in the Hospital Universitario Central de Asturias (HUCA). It included patients diagnosed with psoriasis vulgaris who, on 30 November 2020, were receiving treatment with adalimumab, ustekinumab, secukinumab, or ixekizumab. The study was approved by the Ethics and Research Committee of the Principality of Asturias, Spain (n° 2020.470).

The study included 300 patients in the care of the Dermatology Department. The records of all patients were reviewed and the following data obtained from each patient’s clinical history: sex, age (years), weight (kg), height (cm), family history of psoriasis (a positive family history was concluded if there was psoriasis in at least one first-degree relative), age of onset of skin pathology, whether the patient had received previous treatments (“non-naive”) or not (“naive”), and the presence of arthritis (as diagnosed by a rheumatologist). Comorbidities such as high blood pressure, diabetes mellitus (DM), and dyslipidaemia were identified. The medications taken were reviewed, coding the patients as having hypertension, DM, or dyslipidaemia if they declared it, if it was evident from their clinical history, or if they were taking antihypertensive, antidiabetic, or lipid-lowering medication. Patients whose blood pressure was >135/85 mm Hg, measured during their consultation, were also classified as hypertensive.

The analytical determinations in the medical records were reviewed. Patients with hypertriglyceridaemia (triglycerides >150 mg/dL), hypercholesterolaemia (total cholesterol >200 mg/dL), low-density hyperlipidaemia (LDL >160 mg/dL), were considered to have dyslipidaemia. Body mass index (BMI) was calculated as weight (kg)/square of height (m). Following World Health Organization (WHO) guidelines, values of BMI ≥ 30 were taken to indicate obesity.

The characteristics of the patient cohort are summarized as total frequencies and percentages.

Survival (retention rate) was retrospectively calculated as the time until definitive treatment interruption after initiation treatment.

The patients were divided into two groups: under 65 years of age and 65 years or older (65+) at the time of the study.

### Statistical Analysis

Data were summarised and statistical analyses performed with IBM SPSS version 27.0 (IBM Corp., Armonk, NY, USA). Data are presented as the mean ± standard deviation for continuous variables, and the number and percentage for categorical variables. Group differences for qualitative variables were investigated with the chi-square test. Survival curves were derived using the Kaplan–Meier estimator and compared using the long-rank test. Cox proportional hazard regression models were used for multivariate analyses, and unadjusted and adjusted hazard ratios (HR) were both used to summarize the studied differences. 95% confidence intervals (95% CIs) are also provided. The proportionality of the risks was checked beforehand using the Schoenfeld residual.

We selected the following variables as possible predictors: sex, age of onset of psoriasis, family history, presence of obesity, arthritis, arterial hypertension and dyslipidaemia, and previous use of biological drugs. Group differences were considered statistically significant for values of *p* < 0.05.

The final sample size was sufficient to enable hazard ratios >1.75, proportional differences >25% and standardized differences of means >0.5 to be considered significant (Type I error = 0.05, Type II error = 0.2).

## 3. Results

### 3.1. Patient Characteristics

The study included 300 patients, all of whom were of White ethnicity. Their characteristics are summarized in Table 1. It is worth highlighting that there were almost twice as many men as women in the sample, a predominance of psoriasis patients with a family history and with early onset, and a large minority with concomitant arthritis.

In the under-65-year age group, family history, age of early onset and positivity for HLA-Cw6 were more frequent (67.1% vs. 40.0%, *p* < 0.001; 87.9% vs. 41.7%, *p* < 0.001; 54.2% vs. 11.8%, *p* < 0.001). The over-65-year age-group exhibited more comorbidities: hypertension, dyslipidaemia, and DM (53.3% vs. 25.0%, *p* < 0.001; 63.3% vs. 41.7%, *p* = 0.004; 35.0% vs. 11.3%, *p* < 0.001). No statistically significant differences were found for any other variable (Table 2).

Overall, adalimumab was prescribed in 37% of patients, ustekinumab in 32.7%, secukinumab in 21.3% and ixekizumab in 9%. No statistically significant differences were found in the proportional use of any of the drugs between the two age groups. The distributions of the use of these drugs in the under-65 and 65+ year age groups were adalimumab, 36.7% vs. 38.3%; ustekinumab, 32.1% vs. 35.0%; secukinumab, 22.5% vs. 16.7%; ixekizumab 8.8% vs. 10.0%.

### 3.2. Drug Survival

**Figure 1:**
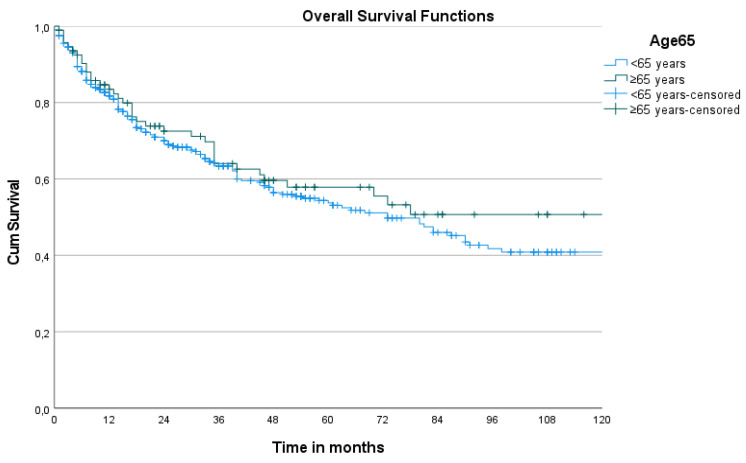
Kaplan–Meier curves of overall survival according to age (*p* = 0.336).

The survival curves for all the biological drugs are similar for the two age groups (Figure 1).

**Figure 2:**
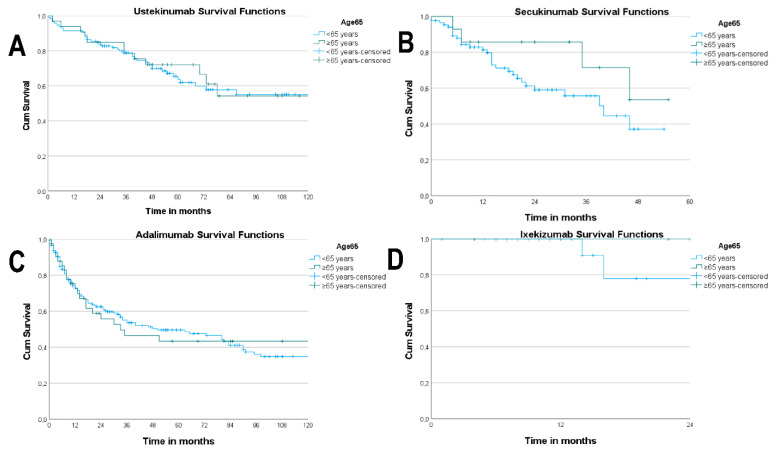
(**A**) Kaplan–Meier curves of ustekinumab survival according to age (*p* = 0.848). (**B**) Kaplan–Meier curves of secukinumab survival according to age (*p* = 0.182). (**C**) Kaplan–Meier curves of adalimumab survival according to age (*p* = 0.761). (**D**) Kaplan-–Meier curves of ixekizumab survival according to age (*p* = 0.494).

The survival curves of the different drugs did not differ significantly between the two age groups (Figure 2A–D).

### 3.3. Multivariate Analysis

The factors predicting the overall lower survival of biological drugs in the multivariate analysis were: being female, obesity, having undergone previous biological treatment and suffering from arthritis. The first three factors were influential in the under-65-year-old group, while arthritis was a significant factor for the older group (Table 3).

With respect to ustekinumab, in the multivariate analysis, suffering from arthritis was a risk factor for discontinuing treatment overall and in the under-65 and 65+ year age groups. Regarding secukinumab, in the multivariate analysis, obesity was the only factor predisposing to discontinuing treatment overall, although this factor, and having undergone previous treatment, was only significant in patients younger than 65 years of age. Turning to adalimumab, the multivariate analysis showed that, overall, being a woman, obesity and having undergone a previous treatment with a biological drug predisposed to suspending treatment. These risks were present in the under-65-year age group, but not in those aged 65 years or more. Too few prescriptions for ixekizumab were issued to allow meaningful statistical analyses to be carried out for this drug (30 treatment cycles, 24 in those under 65 years and 6 in those 65+ years of age).

## 4. Discussion

In accordance with the WHO definition of the elderly, we chose the age of 65 as the cut-point to define the age groups [18]. This age threshold has been chosen in almost all therapeutic studies of older patients with psoriasis, except for one study each that used ages of 70 years [31] and 75 years [32].

Due to the improvement in life expectancy, the prevalence of psoriasis is increasing in the elderly [22]. It is estimated that around 10% of patients with psoriasis belong to this group and 15% of these have moderate–severe psoriasis. There is little information regarding the presentation and treatment of psoriasis in this age group [4], mainly because these patients are excluded from clinical trials because they are considered to be at high risk of suffering adverse effects [19,33].

In our study, 20% of patients receiving a biological treatment were aged 65 years or more, a higher percentage than that in the Spanish national Biobadaderm registry (11%) [34], and in a French study (5%) [4], and slightly lower than that reported by Garber et al. (25%) [35].

Similar to what was previously reported, and as expected, the group of patients over 65 years of age were more likely to exhibit comorbidities, such as hypertension, dyslipidaemia and diabetes mellitus, than the under-65-year age group. Previous epidemiological studies have identified sex- and obesity-related differences between the two groups. We did not find such differences in our series, which may be attributed to the fact that our study considered only elderly patients undergoing biological treatment [3]. CVR factors such as obesity, hypertension, dyslipidaemia, diabetes mellitus and metabolic syndrome were more prevalent in patients with psoriasis [36], which is an independent risk factor for the appearance of these comorbidities [10,23,37,38,39,40,41]. More than 50% of the patients in our series had more than three comorbidities. The frequency of comorbidities in people aged 65+ years was very similar to that reported in the study by Phan et al. [36]. It is important to note the more frequent presence of arthritis in older patients in our series compared with what has previously been published [4,36].

Family history was a more prevalent factor in younger patients, consistent with other studies [3]. We found a higher percentage of HLA-Cw6 positivity in young patients, which partly explains the greater family predisposition in this group [42].

We found no differences between the two groups for the prescribed biological drugs. Our results from patients aged 65+ years were similar to those of Ricceri and co-workers, and as seen in real clinical practice: 75% of their patients were treated with adalimumab, ustekinumab or secukinumab [32].

In our population, the overall survival of all the drugs, combined and separately, did not differ between the study groups, which implies that the efficacy and presence of adverse effects were the same in both groups, similar to what was reported in two systematic reviews of the use of biological drugs in the elderly [26,43].

At the individual level, the same degrees of efficacy and safety have been found in older patients treated with ustekinumab, adalimumab, secukinumab and ixekizumab [44,45,46,47,48].

We are aware of only one study of people over 65 years of age in which survival curves indicated survival rates in the first year around of 80% for secukinumab, ixekizumab, and brodalumab [36], similar to our results. The differences between our patients were minimal in secukinumab and in ixekizumab, but the number of patients in both series was too low to permit statistical comparison. Although we did not find any differences in the overall survival of the biological drugs between the two age groups, it is interesting to note that the variables predicting the discontinuation of biological drugs in people under 65 years of age were different from those pertaining in the 65+ year age group. Thus, globally (under 65 and 65+ years) and in other registries, it has been pointed out that female sex, obesity, the presence of arthritis, and their previous use are the reasons for deciding to discontinue treatment with several biological drugs [49,50,51,52]. With ustekinumab, we found that arthritis was the only factor that reduced the survival of the drug in both age groups, similar to what was concluded from the Badbir study [49]. For secukinumab, obesity was the only factor signalling the need to discontinue treatment, globally and specifically in those under 65 years of age, in line with two previous studies [53,54]. The small number of patients aged 65+ years prevented us from carrying out a meaningful statistical study, as with ixekizumab. In adalimumab, globally and in the under-65-year-old group, we found that being a woman, obesity and having undergone previous treatments reduced the survival of the drug, which is a similar finding to that of the largest series published to date [55]. They were not found to be significant risk factors in patients aged 65 years or more.

Our study has several limitations. The first is that it is an observational study, and therefore prone to the effects of the biases inherent to this type of study. Second, in routine clinical practice, the selection of treatments for each patient is not randomized. In addition, new active treatments of psoriasis have been added that can modify the survival of older drugs. Lastly, our study featured very few patients who received secukinumab and ixekizumab.

## 5. Conclusions

We found that the pattern of prescription of biological drugs to patients and the survival of biological drugs were very similar in patients aged less than 65 years and those aged 65+ years. The risk of discontinuing biological treatment in patients aged 65+ years was associated with the presence of arthritis, while in the group under 65 years of age it was associated with being a woman, obesity and having previously received biological treatments.

## Figures and Tables

**Table 1:** Baseline characteristics of patients.

Total Patients (*n* = 300)	
Sex (male), *n* (%)	189 (63%)
Median age	52.6 ± 13.6
Age ≥65	60 (20.0%)
Positive family history of psoriasis (yes), *n* (%)	185 (61.7%)
Onset before 40 years of age (%)	236 (78.7%)
Duration of disease (months); mean ± SD; median	26.58 ± 13.5; 27
Cw6-positive (*n* = 152)	68 (44.0%)
Comorbidities, *n* (%)	
Obesity (BMI ≥30)	114 (38.0%)
Diabetes mellitus	48 (16.0%)
Arterial hypertension	92 (30.7%)
Dyslipidaemia	138 (46.0%)
Arthritis	124 (41.3%)
Prior treatments with biologics (%)	137 (45.7%)

**Table 2:** Characteristics of patients aged under 65 and 65+ years, noting degree of statistically significant differences (*p*).

	<65 Years	≥65 Years	*p*
Age	47.8 ± 10.6	71.5 ± 5.4	<0.001
Male/Female, *n* (%)	153 (63.8)/87 (36.3)	36 (60.0)/24 (40.0)	0.654
Family history, No/Yes, *n* (%)	79 (32.9)/161 (67.1)	36 (60.0)/24 (40.0)	<0.001
Early/late onset, *n* (%)	211 (87.9)/29 (12.1)	25 (41.7)/35 (58.3)	<0.001
Cw6, positive/negative, *n* (%)	64 (54.2)/54 (45.8)	4 (11.8)/30 (82.2)	<0.001
Evolution time ± SD	25.8 ±13.2	29.6 ± 14.9	0.077
Naive, Yes/No, *n* (%)	135 (56.1)/105 (43.9)	28 (46.7)/32 (53.3)	0.196
Obesity, No/Yes, *n* (%)	149 (62.1)/91 (37.9)	37 (61.7)/23 (38.3)	0.928
Dyslipidaemia, No/Yes, *n* (%)	140 (58.3)/100 (41.7)	22 (36.7)/38 (63.3)	0.004
Diabetes mellitus, No/Yes, *n* (%)	213 (88.8)/27 (11.3)	39 (65.0)/21 (35.0)	<0.001
Arterial hypertension, No/Yes, *n* (%)	180 (75.0)/60 (25.0)	28 (46.7)/32 (53.3)	<0.001
Arthritis, No/Yes, *n* (%)	140 (58.3)/100 (41.7)	36 (60.0)/24 (40.0)	0.884

**Table 3:** Relative risk of all treatments globally, ustekinumab, secukinumab and adalimumab in the older and younger age groups (Cox regression). * The low number of patients in the over-65-year age secukinumab group prevents the Cox analysis from being performed.

	All Biological Drug Treatments								
	Global (*n* = 517)			Under 65 Years (*n* = 418)			65+ Years (*n* = 99)		
	RR	95% CI	*p*	RR	95% CI	*p*	RR	95% CI	*p*
Sex	1.398	1.053–1.855	0.020	1.517	1.106–2.081	0.010	1.323	0.679–2.756	0.411
Obesity	1.668	1.258–2.212	0.000	2.003	1.462–2.745	0.000	0.759	0.383–1.502	0.428
Non-naive	1.373	1.032–1.827	0.030	1.495	1.093–2.046	0.012	0.872	0.432–1.758	0.702
Arthritis	1.472	1.096–1.977	0.010	1.365	0.990–1.880	0.057	2.802	1.268–6.190	0.011
	**Ustekinumab**								
	Global (*n* = 150)			Under 65 years (*n* = 117)			65+ years (*n* = 33)		
	RR	95% CI	*p*	RR	95% CI	*p*	RR	95% CI	*p*
Sex	0.982	0.550–1.756	0.952	0.991	0.499–1.967	0.980	1.113	0.328–3.780	0.864
Obesity	1.076	0.606–1.910	0.803	1.275	0.649–2.508	0.480	0.607	0.151–2.440	0.482
Non-naive	1.374	0.728–2.596	0.327	1.548	0.753–3.182	0.235	0.644	0.148–2.798	0.557
Arthritis	2.591	1.403–4.788	0.002	2.158	1.085–4.290	0.028	6.716	1.310–34.433	0.022
	**Secukinumab**								
	Global (*n* = 100)			Under 65 years (*n* = 86)			65+ years (*n* = 14)		
	RR	95% CI	*p*	RR	95% CI	*p*	RR	95% CI	*p*
Sex	0.751	0.363–1.553	0.439	0.907	0.419–1.959	0.803			
Obesity	2.248	1.094–4.617	0.027	2.541	1.188–5.433	0.016		*	
Non-naive	2.341	0.954–5.743	0.063	2.822	1.072–7.425	0.036			
Arthritis	1.626	0.768–3.443	0.204	1.506	0.705–3.217	0.291			
	**Adalimumab**								
	Global (*n* = 237)			Under 65 years (*n* = 191)			65+ years (*n* = 46)		
	RR	95% CI	*p*	RR	95% CI	*p*	RR	95% CI	*p*
Sex	1.768	1.214–2.574	0.003	1.970	1.291–3.007	0.002	1.296	0.522–3.216	0.576
Obesity	1.790	1.233–2.600	0.002	2.113	1.393–3.206	0.000	0.946	0.377–2.374	0.905
Non-naive	1.632	1.130–2.358	0.009	1.733	1.157–2.596	0.008	1.482	0.568–3.864	0.421
Arthritis	1.023	0.700–1.496	0.906	1.025	0.674–1.559	0.907	1.221	0.429–3.473	0.708

## References

[1] OMIM Clinical Synopsis—#177900—PSORIASIS 1, SUSCEPTIBILITY TO; PSORS1. https://www.omim.org/entry/177900.

[2] Griffiths C.E., Barker J.N. (2007). Pathogenesis and clinical features of psoriasis. Lancet.

[3] Boehncke W.-H., Schön M.P. (2015). Psoriasis. Lancet.

[4] Phan C., Sigal M.-L., Estève E., Reguiai Z., Barthélémy H., Beneton N., Maccari F., Lahfa M., Thomas-Beaulieu D., Le Guyadec T. (2016). Psoriasis in the elderly: Epidemiological and clinical aspects, and evaluation of patients with very late onset psoriasis. J. Eur. Acad. Dermatol. Venereol..

[5] Kim W.B., Jerome D., Yeung J. (2017). Diagnosis and management of psoriasis. Can. Fam. Physician.

[6] Scher J.U., Ogdie A., Merola J.F., Ritchlin C. (2019). Preventing psoriatic arthritis: Focusing on patients with psoriasis at increased risk of transition. Nat. Rev. Rheumatol..

[7] Yamazaki F. (2021). Psoriasis: Comorbidities. J. Dermatol..

[8] Semenov Y.R., Herbosa C.M., Rogers A.T., Huang A., Kwatra S.G., Cohen B., Anadkat M.J., Silverberg J.I. (2021). Psoriasis and mortality in the United States: Data from the National Health and Nutrition Examination Survey. J. Am. Acad. Dermatol..

[9] Hajiebrahimi M., Song C., Hägg D., Andersson T.M.-L., Villacorta R., Linder M. (2020). The Occurrence of Metabolic Risk Factors Stratified by Psoriasis Severity: A Swedish Population-Based Matched Cohort Study. Clin. Epidemiol..

[10] Mehrmal S., Uppal P., Nedley N., Giesey R.L., Delost G.R. (2021). The global, regional, and national burden of psoriasis in 195 countries and territories, 1990 to 2017: A systematic analysis from the Global Burden of Disease Study 2017. J. Am. Acad. Dermatol..

[11] Gottlieb A.B., Dann F. (2009). Comorbidities in Patients with Psoriasis. Am. J. Med..

[12] Takeshita J., Grewal S., Langan S.M., Mehta N.N., Ogdie A., Van Voorhees A.S., Gelfand J.M. (2017). Psoriasis and comorbid diseases: Epidemiology. J. Am. Acad. Dermatol..

[13] Karmacharya P., Chakradhar R., Ogdie A. (2021). The epidemiology of psoriatic arthritis: A literature review. Best Pract. Res. Clin. Rheumatol..

[14] Gladman D.D. (2004). Psoriatic arthritis. Dermatol. Ther..

[15] Mourad A., Straube S., Armijo-Olivo S., Gniadecki R. (2019). Factors predicting persistence of biologic drugs in psoriasis: A systematic review and meta-analysis. Br. J. Dermatol..

[16] Santos-Juanes J., Galache C., Coto-Segura P., Rodrigo L. (2016). The Actual State of Psoriasis Therapies. Psoriasis·Monograph.

[17] Puig L., Carrascosa J.M., Carretero G., de la Cueva P., Lafuente-Urrez R.F., Belinchón I., Sánchez-Regaña M., García-Bustínduy M., Ribera M., Alsina M. (2013). Spanish Evidence-Based Guidelines on the Treatment of Psoriasis With Biologic Agents, 2013. Part 1: On Efficacy and Choice of Treatment. Actas Dermo-Sifiliográficas.

[18] World Health Organization (2016). Proposed Working Definition of an Older Person in Africa for the MDS Project.

[19] Shary N., Kalb R.E. (2020). Optimizing the Treatment of Moderate-to-Severe Psoriasis in Older Adults. Drugs Aging.

[20] Balato N., Patruno C., Napolitano M., Patrì A., Ayala F., Scarpa R. (2014). Managing Moderate-to-Severe Psoriasis in the Elderly. Drugs Aging.

[21] Megna M., Camela E., Cinelli E., Fabbrocini G. (2020). Real-life efficacy and safety of secukinumab in elderly patients with psoriasis over a 2-year period. Clin. Exp. Dermatol..

[22] van Winden M.E.C., van der Schoot L.S., van de L’Isle Arias M., van Vugt L.J., van den Reek J.M.P.A., van de Kerkhof P.C.M., de Jong E.M.G.J., Lubeek S.F.K. (2020). Effectiveness and Safety of Systemic Therapy for Psoriasis in Older Adults: A Systematic Review. JAMA Dermatol..

[23] Ten Bergen L.L., Petrovic A., Krogh Aarebrot A., Appel S. (2020). The TNF/IL-23/IL-17 Axis-Head-to-Head Trials Comparing Different Biologics in Psoriasis Treatment. Scand. J. Immunol..

[24] Singh J.A., Wells G.A., Christensen R., Tanjong Ghogomu E., Maxwell L.J., MacDonald J.K., Filippini G., Skoetz N., Francis D.K., Lopes L.C. (2011). Adverse effects of biologics: A network meta-analysis and Cochrane overview. Cochrane. Database Syst. Rev..

[25] Dávila-Seijo P., Dauden E., Carretero G., Ferrandiz C., Vanaclocha F., Gómez-García F.-J., Herrera-Ceballos E., De la Cueva-Dobao P., Belinchón I., Sánchez-Carazo J.-L. (2016). Survival of classic and biological systemic drugs in psoriasis: Results of the BIOBADADERM registry and critical analysis. J. Eur. Acad. Dermatol. Venereol..

[26] Carrascosa J.M., Notario J. (2014). Supervivencia en terapia biológica. ¿Sabemos a qué nos referimos? ¿Podemos usarla?. Actas. Dermo-Sifiliográficas.

[27] van den Ree J.M.P.A., Kievit W., Gniadecki R., Goeman J.J., Zweegers J., van de Kerkhof P.C.M., Seyger M.M.B., de Jong E.M.G.J. (2015). Drug Survival Studies in Dermatology: Principles, Purposes, and Pitfalls. J. Investig. Dermatol..

[28] Iannone L.F., Bennardo L., Palleria C., Roberti R., De Sarro C., Naturale M.D., Dastoli S., Donato L., Manti A., Valenti G. (2020). Safety Profile of Biologic Drugs for Psoriasis in Clinical Practice: An Italian Prospective Pharmacovigilance Study. PLoS ONE.

[29] Militello G., Xia A., Stevens S.R., Van Voorhees A.S. (2006). Etanercept for the treatment of psoriasis in the elderly. J. Am. Acad. Dermatol..

[30] Dattola A., Silvestri M., Tamburi F., Amoruso G.F., Bennardo L., Nisticò S.P. (2020). Emerging Role of Anti-IL23 in the Treatment of Psoriasis: When Humanized Is Very Promising. Dermatol. Ther..

[31] Momose M., Asahina A., Hayashi M., Yanaba K., Umezawa Y., Nakagawa H. (2017). Biologic treatments for elderly patients with psoriasis. J. Dermatol..

[32] Ricceri F., Bardazzi F., Chiricozzi A., Dapavo P., Ferrara F., Mugheddu C., Romanelli M., Rongioletti F., Prignano F. (2019). Elderly psoriatic patients under biological therapies: An Italian experience. J. Eur. Acad. Dermatol. Venereol..

[33] Carretero G. (2012). Risk of Serious Adverse Events Associated with Biologic and Nonbiologic Psoriasis Systemic Therapy: Patients Ineligible vs Eligible for Randomized Controlled Trials. Arch. Dermatol..

[34] Medina C., Carretero G., Ferrandiz C., Dauden E., Vanaclocha F., Gómez-García F.J., Herrera-Ceballos E., De la Cueva-Dobao P., Belinchón I., Sánchez-Carazo J.L. (2015). Safety of classic and biologic systemic therapies for the treatment of psoriasis in elderly: An observational study from national BIOBADADERM registry. J. Eur. Acad. Dermatol. Venereol..

[35] Garber C., Plotnikova N., Au S., Sorensen E.P., Gottlieb A. (2015). Biologic and Conventional Systemic Therapies Show Similar Safety and Efficacy in Elderly and Adult Patients with Moderate to Severe Psoriasis. J. Drugs Dermatol..

[36] Phan C., Beneton N., Delaunay J., Reguiai Z., Boulard C., Fougerousse A., Cinotti E., Romanelli M., Mery-Bossard L., Thomas-Beaulieu D. (2020). Effectiveness and Safety of Anti-interleukin-17 Therapies in Elderly Patients with Psoriasis. Acta Derm. Venereol..

[37] Sbidian E., Chaimani A., Afach S., Doney L., Dressler C., Hua C., Mazaud C., Phan C., Hughes C., Riddle D. (2020). Systemic Pharmacological Treatments for Chronic Plaque Psoriasis: A Network Meta-Analysis. Cochrane Database Syst. Rev..

[38] Fernandez-Torres R.M., Paradela S., Fonseca E. (2012). Psoriasis in patients older than 65 years. A comparative study with younger adult psoriatic patients. J. Nutr. Health Aging.

[39] Puig L., Kirby B., Mallbris L., Strohal R. (2014). Psoriasis beyond the Skin: A Review of the Literature on Cardiometabolic and Psychological Co-Morbidities of Psoriasis. Eur. J. Dermatol..

[40] Puig L. (2017). Cardiometabolic Comorbidities in Psoriasis and Psoriatic Arthritis. Int. J. Mol. Sci..

[41] Napolitano M., Balato N., Ayala F., Patruno C., Patrì A., Megna M., Balato A. (2016). Psoriasis in elderly and non-elderly population: Clinical and molecular features. G Ital. Dermatol. Venereol..

[42] Chen L., Tsai T.-F. (2018). HLA-Cw6 and Psoriasis. Br. J. Dermatol..

[43] Sandhu V.K., Ighani A., Fleming P., Lynde C.W. (2020). Biologic Treatment in Elderly Patients with Psoriasis: A Systematic Review. J. Cutan. Med. Surg..

[44] Dommasch E.D., Kim S.C., Lee M.P., Gagne J.J. (2019). Risk of Serious Infection in Patients Receiving Systemic Medications for the Treatment of Psoriasis. JAMA Dermatol..

[45] Hayashi M., Umezawa Y., Fukuchi O., Ito T., Saeki H., Nakagawa H. (2014). Efficacy and safety of ustekinumab treatment in elderly patients with psoriasis. J. Dermatol..

[46] Chiricozzi A., Zangrilli A., Bavetta M., Bianchi L., Chimenti S., Saraceno R. (2017). Real-life 9-year experience with adalimumab in psoriasis and psoriatic arthritis: Results of a single-centre, retrospective study. J. Eur. Acad. Dermatol. Venereol..

[47] Körber A., Papavassilis C., Bhosekar V., Reinhardt M. (2018). Efficacy and Safety of Secukinumab in Elderly Subjects with Moderate to Severe Plaque Psoriasis: A Pooled Analysis of Phase III Studies. Drugs Aging.

[48] Megna M., Cinelli E., Balato A., Gallo L., Fabbrocini G. (2020). Efficacy and safety of ixekizumab in a group of 16 elderly patients with psoriasis over a 1-year period. J. Eur. Acad. Dermatol. Venereol..

[49] Yiu Z.Z.N., Mason K.J., Hampton P.J., Reynolds N.J., Smith C.H., Lunt M., Griffiths C.E.M., Warren R.B., the BADBIR Study Group (2020). Drug survival of adalimumab, ustekinumab and secukinumab in patients with psoriasis: A prospective cohort study from the British Association of Dermatologists Biologics and Immunomodulators Register (BADBIR). Br. J. Dermatol..

[50] Kojanova M., Fialova J., Cetkovska P., Dolezal T., Lomicova I., Arenberger P., Gkalpakiotis S., the BIOREP Study Group (2021). Demographic Data, Comorbidities, Quality of Life, and Survival Probability of Biologic Therapy Associated with Sex-specific Differences in Psoriasis in the Czech Republic. Dermatol. Ther..

[51] Menter A., Papp K.A., Gooderham M., Pariser D.M., Augustin M., Kerdel F.A., Fakharzadeh S., Goyal K., Calabro S., Langholff W. (2016). Drug survival of biologic therapy in a large, disease-based registry of patients with psoriasis: Results from the Psoriasis Longitudinal Assessment and Registry (PSOLAR). J. Eur. Acad. Dermatol. Venereol..

[52] Gniadecki R., Bang B., Bryld L.E., Iversen L., Lasthein S., Skov L. (2015). Comparison of long-term drug survival and safety of biologic agents in patients with psoriasis vulgaris. Br. J. Dermatol..

[53] Galluzzo M., Talamonti M., De Simone C., D’Adamio S., Moretta G., Tambone S., Caldarola G., Fargnoli M.C., Peris K., Bianchi L. (2018). Secukinumab in moderate-to-severe plaque psoriasis: A multi-center, retrospective, real-life study up to 52 weeks observation. Expert Opin. Biol. Ther..

[54] Megna M., Di Costanzo L., Argenziano G., Balato A., Colasanti P., Cusano F., Galluccio A.G., Gambardella A., Lembo S., Mozzillo R. (2019). Effectiveness and safety of secukinumab in Italian patients with psoriasis: An 84 week, multicenter, retrospective real-world study. Expert Opin. Biol. Ther..

[55] Kojanova M., Cetkovska P., Strosova D., Fialova J., Arenberger P., Dolezal T., Gkalpakiotis S., BIOREP Study Group (2021). Real-World Evidence from More Than 1000 Patients Treated with Adalimumab for Moderate-to-Severe Psoriasis in the Czech Republic. Dermatol. Ther..

